# Editorial: Microorganisms in sustainable and green agriculture: synergistic effect on carbon sequestration and crop productivity

**DOI:** 10.3389/fmicb.2024.1470240

**Published:** 2024-08-08

**Authors:** Jianling Fan, Yichao Shi, Yunliang Li

**Affiliations:** ^1^Jiangsu Key Laboratory of Atmospheric Environment Monitoring and Pollution Control, Jiangsu Collaborative Innovation Center of Atmospheric Environment and Equipment Technology, School of Environmental Science and Engineering, Nanjing University of Information Science and Technology, Nanjing, China; ^2^Ottawa Research and Development Centre, Agriculture and Agri-Food Canada, Ottawa, ON, Canada; ^3^Department of Soil Science, University of Saskatchewan, Saskatoon, SK, Canada

**Keywords:** soil, microorganisms, root, rhizosphere, inoculants and bio-stimulants

Since the mid-20th century, chemical fertilizers have been widely used to enhance crop productivity, resulting in soil degradation, water pollution, and environmental harm. Microorganisms play a crucial role in soil nutrient cycling and subsequently influence crop productivity, carbon sequestration, soil fertility, and soil health. Specifically, the rhizosphere microbiome could significantly affect plant health, where Plant Growth-Promoting bacteria (PGPB) have emerged as vital allies in mitigating abiotic stresses, such as salt stress and soil-borne diseases. Therefore, it is imperative to develop sustainable and green agricultural systems that can synergistically boost crop productivity, reduce nutrient losses, promote carbon sequestration, improve soil health, and enhance resilience to climate change.

The *Research Topic, Microorganisms in sustainable and green agriculture: synergistic effect on carbon sequestration and crop productivity*, invited contributions in the following areas: (a) The composition and structure of the microbial community and its impact on plant nutrient uptake and soil organic carbon sequestration; (b) Mechanisms of plant-microbe interactions and their effects on nutrient cycling and organic matter turnover; (c) The influence of land use and management practices on the structure and function of soil microbiome and its impact on nutrient uptake and soil carbon storage; (d) The role of microbial inoculants and bio-stimulants in promoting plant nutrient uptake and soil carbon sequestration.

This Research Topic combined a total of nine articles, four of which provide insights into recent advances in soil microbiome response to different agricultural practices, such as the use of inorganic and organic fertilizers, crop rotation and quicklime application, in relation to crop productivity, nutrient uptake, and greenhouse gas emission. These studies covered a broad range of agroecosystems, including cropping systems of maize, peanut, oilseed rape, and grassland. The remaining five articles present new findings in the isolation and functions of PGPB, highlighting that the potential of new isolated strains in *Serratia, Trichoderma, Bacillaceae, Pseudomonas* as beneficial and biocontrol agents in sustainable and green agriculture.

Yang et al. highlighted “*Multi-year crop rotation and quicklime application promote stable peanut yield and high nutrient-use efficiency by regulating soil nutrient availability and bacterial/fungal community*”, wherein a multi-year field experiment was conducted to investigate the effects of crop rotation and quicklime application on peanut nutrient uptake, yield, soil chemical properties, the diversity and function of bacterial and fungal communities. The authors emphasized that wheat-maize-peanut rotation in combination with quicklime application effectively promoted the growth of peanut by improving soil fertility and establishing a healthy soil micro-ecology, thereby mitigating the negative effects of continuous peanut cropping.

Wang et al. investigated “*Synergistic effects of rhizosphere effect and combined organic and chemical fertilizers application on soil bacterial diversity and community structure in oilseed rape cultivation*”, wherein they studied the impacts of different ratios of chemical and organic fertilizers on soil bacterial diversity and community structure, comparing rhizosphere and non-rhizosphere soil in oilseed rape cultivation. This study revealed that a fertilizer mix of 25% chemical and 75% organic significantly increased soil bacterial abundance, diversity, and ecological network complexity, as well as the aboveground biomass of oilseed rape.

Fudjoe et al. assessed “*The impact of fertilization on ammonia-oxidizing bacteria and comammox Nitrospira communities and the subsequent effect on N*_2_*O emission and maize yield in a semi-arid region*”. The findings revealed that fertilization treatments significantly influenced maize productivity, nitrogen use efficiency, and N_2_O emissions. Notably, ammonia-oxidizing bacteria and comammox *Nitrospira* communities exhibited distinct keystone taxa, with both groups substantially contributing to maize productivity, NUE, and N_2_O emissions. The study highlighted that the combined inorganic and organic fertilizer treatment holds considerable promise for reducing N_2_O emissions while enhancing maize productivity.

King et al. compiled their research in a article titled “*Comparative analysis of the soil microbiome and carbohydrate content of Anthoxanthum nitens (Sweetgrass) and other Poaceae grass tissues and associated soils*”, which focused on carbohydrate composition and content in plant tissues of greenhouse-grown Sweetgrass in comparison to other Poaceae grass in the field. They also studied the differences of soil microbial communities across sampling sites.

Furthermore, this Research Topic published five articles related to the isolation and/or functions of PGPB, which are instrumental in enhancing plant resilience and productivity under various stress conditions. Kulkova et al. mini-reviewed “*Serratia spp. as plant growth-promoting bacteria alleviating salinity, drought, and nutrient imbalance stresses*”. These bacteria show promise in promoting plant growth by producing phytohormones, ACC deaminase, fixing nitrogen, solubilizing phosphorus and zinc, enhancing antioxidant properties, and modulating gene expression. This review suggested that further research is needed to understand the molecular mechanisms of *Serratia* spp. and their effects on soil and plant microbiota, utilizing omics techniques.

Liu et al. discussed “*Beneficial and biocontrol effects of Trichoderma atroviride, a dominant species in white birch rhizosphere soil*”, wherein they reported 37 *Trichoderma* strains isolated from rhizosphere soils of White birch (*Betula platyphylla Suk*.), identifying *T. atroviride* as the dominant and most effective biocontrol species due to its stress tolerance and pathogen confrontation abilities. An *in vivo* experiment on Gynura cusimbua seedlings showed that *T. atroviride* enhanced seedling growth, soluble protein and sugar content, and catalase activity while reducing malonaldehyde levels. It also increased soil nitrogen and phosphorus availability and plant nutrient uptake. These traits suggest *T. atroviride*'s potential as a biocontrol agent in agriculture and forestry.

Bao et al. attempted to uncover “*Mechanism on the promotion of host growth and enhancement of salt tolerance by Bacillaceae isolated from the rhizosphere of Reaumuria soongorica*”, wherein three Plant Growth-Promoting Rhizobacteria strains belonging to *Bacillaceae* were isolated from the rhizosphere of *Reaumuria soongorica*. These strains demonstrate tolerance to high salt levels and promoted plant growth by increasing height, biomass, and photosynthetic pigments while reducing stress markers. Strain S40, in particular, was found to reprogram plant metabolism, enhancing hormone signal transduction and promote plant growth under salt stress.

Sun et al. described “*Bacillus velezensis BVE7 as a promising agent for biocontrol of soybean root rot caused by Fusarium oxysporum*”. This study found that *Bacillus velezensis* BVE7, isolated from soybean roots, exhibited broad-spectrum antifungal activity, significantly reducing pathogen growth and the incidence of soybean root rot. The authors emphasized that strain BVE7 enhanced enzymatic activities in soybean roots, boosting plant resistance and reducing disease severity. They highlighted the potential of *Bacillus velezensis* BVE7 as a viable option for soybean root rot management.

Wei et al. discussed “*Pseudomonas chlororaphis IRHB3 assemblies beneficial microbes and activates JA-mediated resistance to promote nutrient utilization and inhibit pathogen attack*”, wherein they studied the effects of IRHB3 on the local rhizosphere microbiome, disease resistance, and soybean growth in a field pot experiment. The authors found that IRHB3 enriched the rhizosphere bacterial community and maintained its balance, even in the presence of *Fusarium oxysporum*. It activated JA-mediated resistance and nodulation genes, thereby enhancing nitrogen fixation and increasing soybean yield.

In conclusion, this Research Topic has provided valuable insights into the complex interactions between microorganisms, agricultural practices, and crop productivity, with a specific emphasis on the critical role of PGPB in sustainable agriculture. The findings from this Research Topic reinforce the importance of integrating microbial and agronomic strategies to develop sustainable and green agricultural systems. Future research should continue to explore these interactions and leverage advanced omics technologies to further understand the molecular mechanisms underlying plant-microbe-soil interactions and their implications for agricultural management.

